# Building a Healthy Future: Functional Motor Skill Development in Precocious Prevention of Childhood Obesity

**DOI:** 10.3390/jfmk10020186

**Published:** 2025-05-22

**Authors:** Debora Porri, Malgorzata Wasniewska, Giovanni Luppino, Letteria Anna Morabito, Elisa La Rosa, Giorgia Pepe, Domenico Corica, Mariella Valenzise, Maria Francesca Messina, Giuseppina Zirilli, Alessandra Li Pomi, Aurora Lanzafame, Valentina Arena, Angela Alibrandi, Debora Di Mauro, Tommaso Aversa

**Affiliations:** 1 Department of Human Pathology of Adulthood and Childhood, University of Messina, Via Consolare Valeria, 98124 Messina, Italy; debora.porri@gmail.com (D.P.); malgorzata.wasniewska@unime.it (M.W.); giorgia.pepe@unime.it (G.P.); domenico.corica@unime.it (D.C.); mariella.valenzise@unime.it (M.V.); francesca.messina@unime.it (M.F.M.); giuseppina.zirilli@polime.it (G.Z.); alessandra.lipomi92@gmail.com (A.L.P.); auroralanzafamemd@gmail.com (A.L.); arena.valentina95@gmail.com (V.A.); tommaso.aversa@unime.it (T.A.); 2 Pediatric Unit, “G. Martino” University Hospital, 98124 Messina, Italy; letteria.morabito@gmail.com (L.A.M.); elisalarosa@icloud.com (E.L.R.); 3 Department of Economics, University of Messina, 98100 Messina, Italy; angela.alibrandi@unime.it; 4 Department of Biomedical and Dental Sciences and Morphological and Functional Imaging, University of Messina, 98100 Messina, Italy

**Keywords:** childhood obesity, childhood obesity prevention, physical activity, lifestyle intervention

## Abstract

Background: The rising prevalence of childhood obesity (CO) has been strongly linked to physical inactivity and sedentary behavior. Early development of functional movement skills (FMS) is crucial for fostering active lifestyles and preventing CO. Methods: We assessed the FMS of 102 children aged 3–5 using the MOBAK test battery. Parents completed a Likert-scale questionnaire evaluating their perception of their child’s motor competence. Results: A total of 102 children and 92 parents participated. Although 61.1% of children engaged in regular sports activities, only 20.5% reached a satisfactory MOBAK total score (Score 3). Significant gender differences emerged in locomotor skills (Score 2) and overall performance (Score 3), with *p*-values < 0.026 and <0.016, respectively. A significant negative correlation between BMI and Score 2 was observed (*p* < 0.030). Parents significantly overestimated their children’s FMS (*p* = 0.0001). Conclusions: Findings emphasize the importance of early interventions targeting FMS enhancement and parental education to effectively support CO prevention strategies and promote lifelong physical activity.

## 1. Introduction

Global and national guidelines on physical activity highlight the importance of daily structured and unstructured physical activity from childhood [1]. Regular physical activity improves physical, mental and cognitive health outcomes, and children and adolescents should limit sedentary time, especially the time spent on the screen for recreational purposes [1,2]. Despite global guidelines, physical inactivity and sedentary behaviors are relevant public health problems of the 21st century [3]. In Italy, our region of study, the prevalence of insufficient physical activity is higher in adolescent girls than boys, with an increasing trend from 2001 to 2016 exceeding 90% for the female gender [4]. Such behaviors often persist into adulthood, showing gender-related disparities, and are frequently rooted in habits established during early childhood [1,2,3,4]. Similarly, overweight and obesity beginning in early childhood tend to persist into later life stages [5].

The overall prevalence of overweight in children is about 8.5%, which means that at least one in five children have excess weight [6,7]. Among the various contributing factors, physical inactivity plays a central role, significantly affecting energy balance and acting as a modifiable determinant of childhood obesity (CO) [7]. Therefore, national and regional health strategies should prioritize the promotion of physical activity from an early age. According to WHO guidelines, children under 5 years should accumulate at least 180 min of physical activity per day, including at least 60 min of moderate-to-vigorous physical activity (MVPA) for children aged 3–5 years [1,4]. Preschool age (3–5 years) is a particularly sensitive period for motor development. It represents a unique opportunity to acquire fundamental movement skills (FMSs), which are the building blocks of more specialized and refined movements required in both organized sports and spontaneous play [8,9]. Childhood is also an ideal time to develop functional movement skills (FMSs) and, later, to refine these motor skills in a specific context and sports practice [10]. In children who do not receive adequate motor practice and instruction, a delay in the development of motor skills and a lower propensity for physical activity have been demonstrated [8]. FMSs are regarded as the foundational elements that lead to specialized movement patterns necessary for effective participation in various organized and unorganized physical activities [9]. Locomotor, manipulative, object control and stability skills, included among the FMSs, are acquired with appropriate practice, instruction and encouragement [9], and proficiency in FMSs should be considered an indicator of the quality of elementary school physical education, as in several countries [8,9,10]. Beyond their physical implications, FMSs are linked to broader developmental outcomes. Higher motor competence in young children has been associated with better cardiorespiratory fitness, muscular strength, and cognitive and social development [11,12,13,14,15]. Specifically, balance skills have shown strong associations with cardiorespiratory fitness, higher physical activity intensities, and a healthier BMI status during adolescence [15].

Several studies investigated the relationships between FMSs and anthropometric parameters both in children and adolescents, and a negative association was found between BMI level and FMSs and motor competencies [16,17,18]. Particularly children with overweight or obesity, such as those less active and more sedentary, present lower scores of FMS development, and BMI is negatively correlated with motor skills, physical activity level, daycare attendance, lower body fat percentage and male sex [17,18,19,20]. FMS development is influenced not only by biological and environmental factors but also by psychosocial elements—particularly the role of parents [21]. As key influencers of young children’s routines, parents shape physical activity through both support and their perception of motor skills. However, they often overestimate these abilities, potentially overlooking delays and missing chances for early intervention [22,23,24]. This highlights the importance of increasing parental awareness and providing guidance to support accurate assessment and effective promotion of motor competence during early childhood.

The present study is part of the “0–6 EpPoi–Educare per Prevenire l’Obesità Infantile” project, which promotes early, multi-sectoral strategies to prevent childhood obesity, including reducing sedentary behavior [25]. In previous research, 79.2% of respondents gave the highest rating (5 on a Likert scale) to the question, “*How useful do you think it is to talk about preventing childhood obesity through lifestyle [nutrition and physical activity]?*” [25,26].

In addition, 62.5% of interviewed subjects declare that they would like to have more information about physical activity during childhood [26]. A key focus of the 0–6 EpPOI project is implementing recreational and physical activity programs with local administrations to promote healthy lifestyles and prevent early childhood obesity. This cross-sectional study primarily aims to assess fundamental motor skills (FMSs) in preschoolers aged 3–5 to support targeted interventions. A secondary aim is to explore parents’ perceptions of their children’s physical abilities, helping align awareness with actual skills and guide caregiver-inclusive strategies.

## 2. Materials and Methods

### 2.1. Selection Criteria and Data Collection

The target population was children from 3 to 5 years of age, of both sexes and any ethnicity, and their families. Thanks to the strong collaboration previously established with the school, we were able to recruit a representative and randomly selected sample of children from each age group between 3 and 5 years.

The study was conducted in a preschool in Messina, Italy, and it is being conducted by a multidisciplinary team on dedicated days during the school timetable for data recording. The anonymous online survey for parents was administered by means of Google Forms and data were downloaded as a Microsoft Excel sheet.

Participants were fully instructed about the study aim and were also informed that by agreeing to fill in the questionnaire, they confirmed their participation, automatically providing informed consent. There were no direct benefits to the respondents from participating in this study.

### 2.2. MOBAK Test and Physical Activity Evaluation

A kinesiologist with expertise in childhood physical activity performed the MOBAK test to assess each child’s effective FMSs. This battery of 8 tests measures object movement, such as throwing, catching, bouncing, and dribbling, and self-movement, such as balancing, rolling, jumping, and running. Both subscales have a maximum of 8 points, producing a combined MOBAK score of 0 (lowest) to 16 (highest). Four items (throwing, catching, bouncing, and dribbling) cover the basic motor competency “*object control*”, while the remaining four (balancing, rolling, jumping, and sidestepping) cover the basic motor competency “*locomotion*” [27].

To assess parents’ perception of their child’s lifestyle, information on any structured sport activities and sleep habits, we performed an online survey; furthermore, we investigated parents’ perception of the motor functions investigated with the MOBAK test. The survey is made up of 5 questions in the form of a Likert scale where 1 indicates “a little” and 5 indicates “a lot”, with the addition of some demographic information. Specific motor skills (such as running, jumping, and walking on an unstable surface) are important indicators of a child’s physical development [28], so we asked parents whether they thought their children could perform these activities. The complete record of questions proposed is listed in Table 1.

### 2.3. Anthropometric Parameters

Children’s weight and height were measured with standardized procedures by a trained pediatrician. Body weight was measured wearing only clothes and without shoes by using a portable electronic scale; height was measured without shoes by means of a portable stadiometer. Waist circumference was measured to the nearest centimeter with a flexible steel tape with children standing with crossed arms and placing the hands on opposite shoulders; waist circumference was measured on the horizontal plane between the lowest portion of the rib cage and the uppermost lateral border of the right ilium [25].

BMI was calculated as a ratio between weight in kilograms and height squared in meters. The waist-to-height ratio was calculated as a ratio between waist in centimeters and height in centimeters.

## 3. Statistical Analysis

Categorical variables were expressed as absolute frequencies and percentages, and the numerical variables as mean ± standard deviation [SD], minimum and maximum.

A radar chart was made to show the average satisfaction score of the respondents for each item [measured on a Likert scale] investigated through the formulation of questions.

To perform a comparison between male and female subjects, the Student *t*-test was applied with reference to numerical variables (scores); in order to compare the proportion between subgroups of patients, the z-test was applied. In addition, a histogram (with a normal curve) was created to show the distribution of the scores. All statistical analyses were performed by using SPSS for Windows, version 22.

## 4. Results

The total number of children participating in the study was 102; 47.1% were male and 52.9% were female. Mean age was 4.41 ±  0.49 years. Mean weight was 19.26 ± 3.80 Kg, mean height was 1.08 ± 0.05 cm, and, thus, mean BMI was 16.17 ± 2.43 Kg/m2. Mean waist circumference was 49.59 ± 5.63 cm with a mean waist-to-height ratio of 0.45 ± 0.04 cm, even if 15.68% of children had a waist-to-height ratio greater than 0.5.

**Figure 1:**
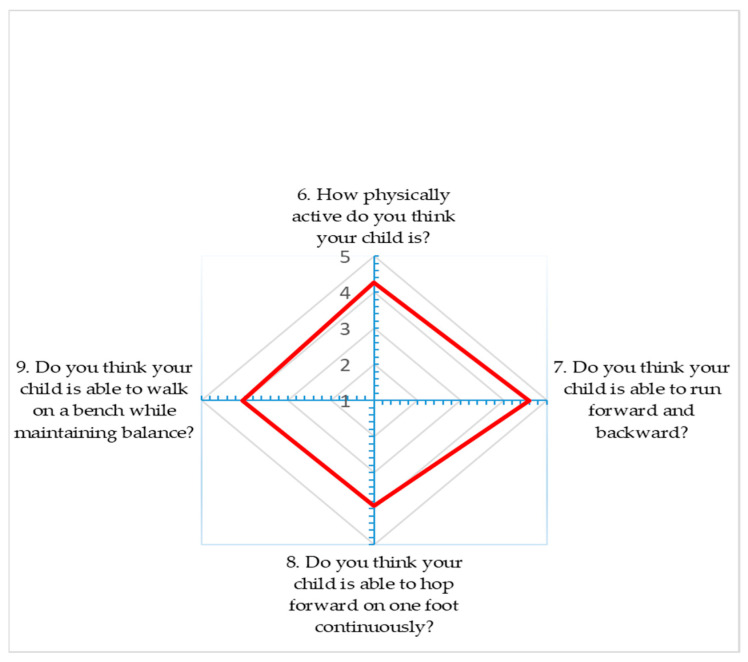
Radar chart.

Ninety-two parents completed the survey, and it is important to underline that they were all mothers; no fathers completed the survey. The percentage frequencies relating to demographic variables (age and level of education) of respondents are summarized in Table 2. Results from questions investigating the Likert scale are reported in Table 3, expressed as frequency and percentage and results are also expressed as a radar chart (Figure 1).

More than half of the mothers declared that their child regularly practices a sport (61.1%), while 38.5% do not play any activity. Among children who do sports regularly, the most popular sports are dance and football, at 12.5% and 7.3%, respectively.

Regarding the FMS evaluation, we added the scores for each of the two MOBAK sections, obtaining *Score* 1 for the “*object control*” section and *Score* 2 for the FMSs in section “*locomotion*”. The sum of the entire questionnaire items was identified as *Score* 3.

The average *Score* 1 was 2.67 ±  1.78, while the *Score* 2 was 4.96 ±  1.96 (means ±  standard deviation).

**Figure 2:**
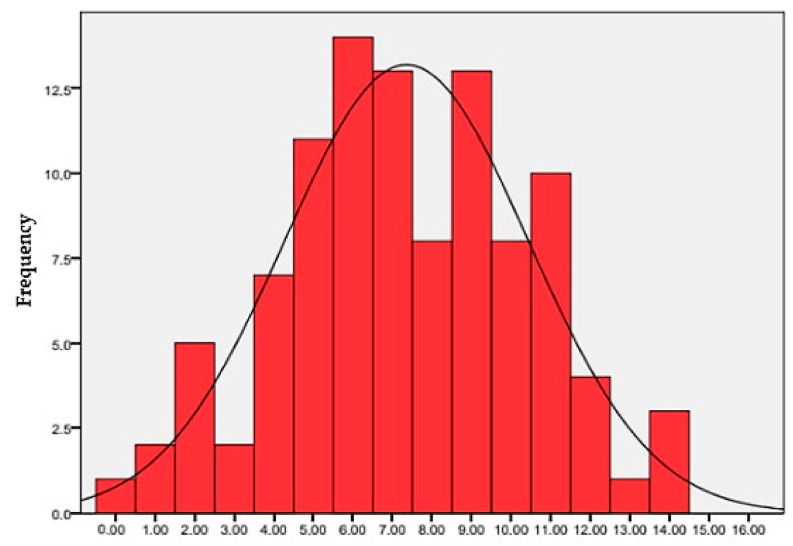
Histogram (with normal curve) for Score 3 distribution.

Only 20.5% of children achieve a *Score* 3 ≥ 8, half of the maximum achievable score, and none reach the maximum score of 16. The maximum *Score* 3 achieved is 14, but only 2.9% of children reach this score. Figure 2 graphically represents the distribution of the percentage frequencies of the *Score* 3 obtained by the children under examination.

We analyzed the sample by dividing it into males and females, and the results of the scores obtained are summarized in Table 4. We found a statistically significant difference comparing males and females, and, in particular, males obtained a significantly higher *Score* 2 and *Score* 3 than females (*p* < 0.026 and *p* < 0.016, respectively).

Interestingly, considering weight status in the entire sample, we found a significant negative correlation between BMI and *Score* 2 (*p* < 0.030) but no other significant correlation between BMI and *Score* 1 and *Score* 3 (*p* < 0.916; *p* < 0.192).

Finally, we evaluated whether parents’ perceptions aligned with their children’s FMSs. We asked parents to estimate their child’s ability to perform specific basic motor activities assessed by the MOBAK test (*Do you think your child is able to run forward and backward? Do you think your child is able to hop forward on one foot continuously? Do you think your child is able to walk on a bench while maintaining balance?* (see Table 3)). The average parental perception score was calculated as 83.62%, derived by summing the scores and dividing by a maximum value of 15.

Similarly, we calculated the children’s *Score* 2, obtaining an average of 58.58% by adding the scores and dividing by the total of 8. This allowed us to compare the two values through a proportions comparison (Table 5), revealing a statistically significant difference between parents’ perceptions and children’s actual FMSs.

## 5. Discussion

Encouraging the development of basic motor skills in preschool children is important because of the direct connection with physical health, academic performance and psychosocial well-being [29]. In addition, school could represent an opportunity to monitor levels of sports performance and to structure tailored activities aimed at developing basic motor competencies.

The evaluation of FMSs in our sample is discouraging; no one reaches the maximum score of 16, and only 20.5% of the children reach a score equal to or higher than half of the maximum score obtainable, showing that they have not yet fully developed the motor skills investigated, despite this being a crucial age period to consolidate as physically competent [30].

Similar results were shown in a recent trial [31] aimed at evaluating the progression of FMSs over a year in children aged 4–5 years at a health center. Despite statistically significant improvements in the total MOBAK test score, the children had not yet reached the halfway point of the total score, confirming the widespread underdevelopment of motor skills in this age group despite potential growth windows.

Recent data from the Italian National Surveillance System [32] evaluating over 46,000 children in primary school reveal that only 39.3% practiced sports 2 days a week and 21.8% practiced sports 3 days a week; in addition, data showed an altered perception: 59.6% of mothers of physically inactive children believed that their child performed adequate physical activity [32]. In our sample, the percentage of children who regularly practice sports was higher (61.1%), but data concerning mothers’ perceptions was similar to previously mentioned data [32,33]: we found a statistically significant difference between parents’ perceptions and children’s actual FMSs, which was not satisfactory as discussed above.

Parents greatly overestimate their children’s abilities, and while they understand that physical activity is crucial for preventing childhood obesity, they may not recognize that their children’s actual activity levels are insufficient, which can contribute to excessive weight gain. As our study shows, parents are not aware of the lack of development of their children’s skills, as also highlighted by Flynn et al. [34], who explain the lack of knowledge on the part of parents to improve children’s FMSs. Parents did not know that these skills should be stimulated and that they could be learned at such a young age. In the study conducted by Wick et al. [35], parents assumed that motor skills such as hopping, throwing, or balancing would naturally emerge with age, without the need for structured support. This misconception may contribute to missed opportunities for early interventions and underlines the importance of equipping caregivers with knowledge to actively foster FMS development. Evidence implied the need to improve the exchange of information necessary between professionals and parents to ensure a correct development of motor skills. This, when combined with the poor development of FMSs in children, may represent an important area of childhood obesity prevention, and school should become a strategic environment in which to focus on children’s motor skills already at preschool age. An intervention study [36] evaluating the effects of regular physical education in preschool settings showed that children aged 4–5 years who participated in structured motor sessions twice per week made significant gains in balance, coordination, and object control skills compared to peers in the control group. These findings highlight the potential of school-based programs in improving physical competence during a developmental stage critical for establishing lifelong physical activity habits [35,36,37].

Regarding ponderal status, we found a significant negative correlation between BMI and *Score* 2, which includes locomotor skills such as balance, rolling, jumping and side-stepping and similar results were shown in a recent multinational study in which the authors examined the association between motor skill competence and BMI in a sample of 5545 preschool children [17]. They found negative associations of locomotor skills and other associations (ball skills and overall motor skill competence) that are probably not evident in our sample due to the small sample size. The negative association between BMI and physical activity levels is well known [38,39], but with a thorough knowledge of the motor skills most involved in this relationship, it is possible to design more specific and targeted interventions. Results from a systematic review conducted by Engel et al. [12] revealed that training FMSs in preschoolers at least three times a week can increase the intensity of physical activity, significantly contributing to reducing childhood obesity risks, and this contribution could be greater if more specific.

Interestingly, we also found that males obtained a significantly higher *Score* 2 than females, possibly suggesting a gender difference in the development of FMSs. In the study conducted by Praxedes et al. [16], an ordinal multilevel logistic regression was performed to analyze the associations of weight status, physical activity, sedentary time and socioeconomic status with FMSs, adjusted for sex and age in a sample of 1014 children aged 6–10 years old, and results revealed that boys were more likely to achieve higher FMS scores.

According to evidence, it is likely that there is a wide range of individual differences in the development of motor competence between different ages and genders, but these differences can be modified [40,41]. As previously mentioned [5], the prevalence of insufficient physical activity in Italy is higher among girls than boys, which could be partly attributed to a lack of FMS development. This observation may suggest the need to focus greater attention on this group, particularly at an early age, to help reduce sedentary behaviors and to prevent overweight.

The deficits found in early childhood, due to the lack of stimulation of basic motor skills, will be evident in adolescence. FMS development in children and adolescents is often dependent on the quality of the instructional environment and the provision of practice-based opportunities, augmenting the importance of key stakeholders, such as physical education teachers, sport pedagogues, coaches, and researchers, within this process [32,34] who should collaborate with clinicians. Improvements in FMSs occur both with specific rules of play, such as the size of the play area, number of participants, etc., and through free play, which also seems to lead to improvement in these skills. However, the presence of a teacher/trainer has been shown to improve learning by encouraging the child to explore alternative movement solutions. It would be useful to increase physical activity levels at school with interactive and innovative lessons structured on the needs of children in relation to their age and motor skills, with a dual role that could promote both the development of FMSs and the development of cognitive functions [42,43].

Moreover, it is worth noticing that this study presents some limitations; for example, due to the small sample size, we could not provide information on the differences between motor skills in children with overweight/obesity compared to normal-weight children, and this comparison would be interesting to conduct a more in-depth analysis.

In this 0–6 EpPOI project satellite study, we focused on the importance of physical activity in preschool age, particularly on the importance of developing FMSs. The implementation of childhood obesity prevention programs focused on physical activity is a well-known consideration, but knowing in detail the aspects related to physical activity in which children are deficient or that could negatively influence excess weight could ensure greater effectiveness. Further studies are therefore needed to clarify the best ways to promote the development of FMSs in order to structure physical activity-tailored programs for childhood obesity prevention.

We finally summarized general practical advice for parents and professionals based on our experience in carrying out the 0–6 EpPOI project, despite there being no standardized guidelines for the development of FMSs that analyze load parameters such as frequency, intensity, duration and mode of administration. Our study could serve as a starting point for further investigations into the topic, ultimately leading to the development of evidence-based clinical practices in the field of physical activity as follows:

- Increase time for physical education at school and carry out scheduled tests to assess FMS development.
- Carry out activities related to improving FMSs in the family environment from an early age.
- Increase free play/play with rules and manipulation of objects during free time.
- Improve social spaces (courtyards, playgrounds, etc.) to provide safe spaces for physical activity.
- Have at least two active breaks of 5/10 min during lessons to reduce sedentary time at school.

To enhance the translational value of the recommendations, age-specific intervention frameworks should be considered, and programs should be complemented by regular assessments to tailor interventions to individual developmental needs. Collaboration between educators, healthcare professionals, and parents is essential for creating an environment that fosters motor skill development and reduces childhood obesity risk, ensuring long-term physical activity habits.

## Figures and Tables

**Table 1:** Complete questionnaire.

Question	Answer
1. Are you the mom or the dad?	Mom; Dad
2. Age group	>20
	20–25
	25–30
	30–35
	35–40
	>40
3. Education level	primary school diploma
	middle school diploma
	diploma
	degree
4. Does your child participate in any sports activities? (swimming, soccer, dance…)	Yes; No
5. If so, which one?	
6. How physically active do you think your child is?	form 1 [a little] to 5 [a lot]
7. Do you think your child is able to run forward and backward?	form 1 [a little] to 5 [a lot]
8. Do you think your child is able to hop forward on one foot continuously?	form 1 [a little] to 5 [a lot]
9. Do you think your child is able to walk on a bench while maintaining balance?	form 1 [a little] to 5 [a lot]

**Table 2:** Parents’ demographic characteristics expressed as frequency and percentage.

Answers	Frequency	Percentage
**Age**		
20–25	5	5.2
25–30	16	16.7
30–35	27	28.1
35–40	25	26.0
>40	22	22.9
**Education level**		
primary school diploma	3	3.1
middle school diploma	52	54.2
diploma	38	396
degree	2	2.1

**Table 3:** Questions investigated by the Likert scale expressed as frequency and percentage.

Likert Scale	Frequency	Percentage
*How physically active do you think your child is?*		
1	1	1.0
2	3	3.1
3	18	18.8
4	19	19.8
5	53	55.2
*Do you think your child is able to run forward and backward*?		
1	0	0
2	2	2.1
3	12	12.5
4	9	9.4
5	71	74.0
*Do you think your child is able to hop forward on one foot continuously?*		
1	7	7.3
2	5	5.2
3	20	20.8
4	19	19.8
5	43	44.8
*Do you think your child is able to walk on a bench while maintaining balance?*		
1	5	5.2
2	5	5.2
3	16	17.0
4	23	24.5
5	45	46.9

**Table 4:** Results from MOBAK test expressed as mean ± SD. *Score* 1: sum of items in Section 1 “*object control*”; *Score* 2: sum of items in Section 2 “*locomotion*”; *Score* 3: sum of *Score* 1 and *Score* 2.

	Gender	*n*	Mean	SD
*Score* 1	M	48	2.97	1.63
	F	54	2.38	1.87
*Score 2*	M	48	5.14	2.01
	F	54	4.27	1.83
*Score* 3	M	48	8.12	3.01
	F	54	6.66	3.00

**Table 5:** Comparison of proportions.

*Difference*	25.04%
*95% CI*	12.48% to 36.48%
*Chi-squared*	14.89
*DF*	1
*Significance level*	*p* = 0.0001

## Data Availability

The datasets presented in this article are not readily available because the data are part of an ongoing study.
